# Predicting In Vivo Efficacy of Potential Restenosis Therapies by Cell Culture Studies: Species-Dependent Susceptibility of Vascular Smooth Muscle Cells

**DOI:** 10.2174/1874192400802010060

**Published:** 2008-07-30

**Authors:** Epstein Hila, Laura Rabinovich, Shmuel Banai, Vicktoria Elazar, Jianchuan Gao, Michael Chorny, Haim D Danenebrg, Gershon Golomb

**Affiliations:** 1 Dept. of Pharmaceutics, School of Pharmacy; 2 Dept. of Cardiology, Bikur Cholim Hospital; 3 Dept. of Cardiology, Hadassah Hospital, Faculty of Medicine, The Hebrew University of Jerusalem, Jerusalem 91120, Israel

**Keywords:** Angioplasty/coronary intervention, cell culture / isolation, restenosis, smooth muscle cells.

## Abstract

Although drug-eluting stents (DES) are successfully utilized for restenosis therapy, the development of local and systemic therapeutic means including nanoparticles (NP) continues. Lack of correlation between *in vitro* and *in vivo* studies is one of the major drawbacks in developing new drug delivery systems. The present study was designed to examine the applicability of the arterial explant outgrowth model, and of smooth muscle cells (SMC) cultures for prescreening of possible drugs. Elucidation of different species sensitivity (rat, rabbit, porcine and human) to diverse drugs (tyrphostins, heparin and bisphsophonates) and a delivery system (nanoparticles) could provide a valuable screening tool for further *in vivo* studies. The anticipated sensitivity ranking from the explant outgrowth model and SMC mitotic rates (porcine>rat>>rabbit>human) do not correlate with the observed relative sensitivity of those animals to antiproliferative therapy in restenosis models (rat≥rabbit>porcine>human). Similarly, the inhibitory profile of the various antirestenotic drugs in SMC cultures (rabbit>porcine>rat>>human) do not correlate with animal studies, the rabbit- and porcine-derived SMC being highly sensitive. The validity of *in vitro* culture studies for the screening of controlled release delivery systems such as nanoparticles is limited. It is suggested that prescreening studies of possible drug candidates for restenosis therapy should include both SMC cell cultures of rat and human, appropriately designed with a suitable serum.

## INTRODUCTION

Percutaneous coronary interventions (PCI, balloon dilation, endoluminal stenting, excisional atherectomy, intravascular brachytherapy, and laser ablation) have revolutionized the treatment of coronary artery disease during the past two decades [1-3] A major recent advancement is the development of local therapy by DES that dramatically reduce the incidence of in-stent restenosis up to <10% [4, 5]. The mode of action of the drugs released from DES, such as sirolimus (rapamycine) and paclitaxel (taxol), is the inhibition of SMC proliferation [6]. Nevertheless, the currently approved DES do not resolve all the problems arising from PCI. There are no DES that are applicable for challenging lesions such as lesions in small arteries, arterial intersection, and multiple injury sites [7]. In recent years, systemic therapies [8-1] and nanoparticles (NP) for systemic and local delivery are being developed [12-14]. The application of an apparently successful drug and/or a delivery system in an animal model of restenosis (rat, rabbit, or porcine) to clinical trials has been frequently encountered with unexpected failures [1, 15-19]. The diverse effects between different experimental models including, tissue culture, animal models of restenosis, and clinical trials is no more evident than with heparin [18, 19].

Heparin has been found effective in various vascular SMC tissue cultures *in vitro* [20, 21], and in the rat and rabbit injury models *in vivo* [18, 22]. Contradicting results were reported in the porcine model [23, 24], it was found ineffective in a primate model [25], as well as in human clinical trials [18, 19].

For the facile development of the next generation of DES, and for the successful implementation of potential systemic as well as local drug delivery treatments, there is a need for a reliable *in vitro* screening method of potential pharmacological agents. We hypothesized that appropriate cell or tissue culture studies can predict, to some extent, the outcome that will be obtained *in vivo,* and consequently in humans.

This study was intended to address the specific questions of: Can cell culture studies of both free drug and drug embedded in a polymer predict the results obtained in animal *in vivo* studies? And to this end, what is the preferred SMC culture species (rat, rabbit, porcine or human) and technique (cell or tissue cultures)? We evaluated the correlation between *in vitro* tissue culture results to that obtained in animal studies by examining several known anti-restenotic drugs of different categories; heparin, tyrphostins and bisphosphonates (BP). Heparin was examined in cell cultures of different species in order to determine whether it's inactivity in the porcine model [24], and in humans [18, 19], following successful* in vitro* [26]**and *in vivo* studies [27-29] could have been predicted by appropriate tissue culture studies. For obtaining an answer to the questions mentioned above, we have examined tyrphostins as a free drug or encapsulated in NP in cell cultures of various species [30, 31]. These low MW and hydrophobic protein tyrosine kinase (PTKs) inhibitors [32], have been shown to inhibit restenosis in various experimental models [12, 33, 34]. In addition, BP, bone-seeking agents, found to be effective in various animal models of restenosis following systemic administration in nanoparticles [35-37] were examined as a negative control. Unlike both heparin and tyrphostins which are effective SMC inhibitors, NP of BP exert their antirestenotic effect by a systemic immunomodulation, with no direct effect on SMC proliferation [35, 36].

## MATERIALS AND METHODS

### Outgrowth Rate of SMC from Arterial Explants

Freshly dissected blood vessels from animals killed less than 5min prior to harvesting or from patients undergoing surgery, were placed in cold Ringer bicarbonate buffer. The arterial explants were used for the outgrowth model and SMC isolation. Animal arterial tissues used were, the abdominal or carotid aorta of Sabra male rats (200-300g); carotid or abdominal arteries of juvenile porcine (15-20kg); and carotid arteries of New Zealand White rabbits (2.5 to 3.5 kg), all of Harlan Laboratories, Jerusalem, Israel. The left internal mammary artery of patients undergoing bypass surgery was utilized. The vessels were aseptically cleaned by removing the fat and connective tissue and were cut longitudinally. The lumen side was gently scraped with a scalpel blade to dislodge endothelial cells, the vessel wall was cut with a scalpel blade into 2x2mm explants, and the luminal surface was gently pressed on fibronectin-coated surface (4 explants/well). Explants were left to adhere undisturbed for 1hr, 1ml of growth media was added and they were incubated for 5 days. In rat explants, the medium contained, 10% fetal calf serum (FCS) high glucose (4500mg/l), penicillin (100U/ml), streptomycin (0.1mg/ml), nystatin (12.5U/ml), gentamycin (50µg/ml) and glutamine (2mmol). Media contained 15% inactivated porcine serum in porcine culture, 15% inactivated rabbit serum in rabbit's culture, and 7.5% FCS and 7.5% human-pooled serum in human’s culture. The explants were placed in a humidifier incubator at 37°C and 8% CO2 atmosphere, and the medium was changed every 2 days. Each explant was scored every 2 days after the first 5 days in order to determine the number of explants yielding a positive outgrowth of SMC. Positive outgrowth was defined as at least 2 cells emerging from the explant border. Scoring was continued until the percentage of explants yielding outgrowth was constant. Explants that had not adhered to the dish were not included in the analysis.

### Inhibition of SMC Outgrowth from Arterial Explants

For studying the inhibition of SMC outgrowth, porcine explants were chosen since they exhibited the fastest growth rate. Medium with or without inhibitors (tyrphostin, AG-1295 (6,7-dimethyl-2-phenylquinoxaline, Oz Chemicals, Jerusalem, Israel), heparin (Sigma-Aldrich, Israel), and bisphosphonates (alendronate (ALN), Unipharm, Tel-Aviv, Israel) was changed every 2 days thereafter. Each explant was scored every two days after the first 5 days as above.

### SMC Isolation and Culture

Rat, porcine, rabbit and human SMC were isolated from explanted arteries and incubated as detailed above. Explants were aspired when the cells reached confluence, cells passages by trypsinization were placed in 5 or 10ml dishes, and the medium was changed every two days. Cells were used from the 1^st^ through the 4^th^ passage. Cells' identity was confirmed by means of microscopic observation and smooth muscle α-actin immunocytochemistry. Unless otherwise noted, all materials for cell cultures were purchased from Biologic Industries (Beit Haemek, Israel) and from Sigma, Israel.

For growth assay, SMC were seeded (day 0) in fibronectin coated 4-multiwell plates (10,000-15,000 cells/well) in the respective growth media. On the following day (day 1), one plate was used to check the number of attached cells, and the medium was changed in the remaining plates with the same medium with or without growth factors and inhibitors. Medium was changed every 2 days, and the cells were counted by means of a Coulter Counter (Coulter Corporation, Miami, FL, USA). For mitotic index determination, the cells’ replication rate was calculated as log_2_ (number of cells 6 days after plating divided by the number of cells 24hr after plating). For serum free medium (SFM) growth curves, cells were seeded in normal growth media at three different densities (low density (LD), 10,000 cells/well, medium density (MD), 30,000 cells/well and high density (HD), 50,000 cells/well). In the following day, the medium was changed to SFM containing transferrin 25mg/ml, insulin 1mg/ml, selenium 1.5*10-8M, cholesterol, vitamin E and taurine with or without serum. Medium was changed every 2 days, and cell number was determined. Proliferation was determined also by using the MTT (3-(4, 5-dimethylthiazolyl-2)-2,5-diphenyltetrazolium bromide) assay.

### Inhibition of SMC Proliferation in Cell Culture

Inhibition of cell proliferation was performed on SMC from passages 2 to 4. SMC were plated at 2x10^4^ cells per well in 24-well plates and were allowed to grow overnight. Inhibitors studied were, tyrphostins (AG-1295, AG-1296, AG-1851, and AG-2033 [32]), heparin, and bisphosphonates (alendronate (ALN), risedronate, and clodronate (clod), Unipharm, Tel-Aviv, Israel, and ISA [2-(2-aminopyri-midino) ethyldiene-1,1-bisphosphonate acid betain], synthesized in our group [38]. The drugs were utilized in their free form, in solution, or encapsulated in NP. Since tyrphostins were diluted in DMSO, the appropriate control used was the normal medium with the respective concentration of DMSO. The cells were then incubated at 37˚C for 48hr, and were counted. In addition, cell viability assay was performed [33, 39].

### Nanoparticles Preparation

#### Bisphosphonate NP

A modified double emulsion-solvent evaporation technique was used to prepare NP of PLGA (poly(lactic-co-glycolic acid)) containing alendronate [37]. Briefly, 0.5 ml of Polyvinyl alcohol, MW 30,000-70,000 (PVA, Sigma-Aldrich, Israel) 2.8% solution in Tris buffer pH 7.4 containing 20 mg alendronate was emulsified in 3 ml of dichloromethane (DCM, J.T. Baker) containing 3% PLGA (Sigma-Aldrich, Israel) by sonication over an ice-bath using a probe sonicator (Microson XL, Misonix, Farmingdale, NY, USA), at 13 W output for 90 seconds. The resulting primary emulsion was added to a 2% PVA (20 ml) solution in Tris buffer pH 7.4 containing CaCl2 (Merck) at a 2:1 molar ratio of Ca to ALN, and sonicated for 90 seconds at 18 W output over an ice bath to form the double emulsion. DCM was eliminated by 3 hr evaporation under magnetic stirring at 4°C.

#### Tyrphostin NP

NP containing the tyrphostin, AG-1295, were formulated using the modified nanoprecipitation method as previously described [33]. Briefly, 200 mg of d,l-PLA poly(d,l-lactide) MW 90,000–120,000 (Sigma, St. Louis, MO, USA) and 2mg of tyrphostin AGL-1295, (Oz Chemicals, Jerusalem, Israel) were dissolved in an organic phase of dichloromethane and acetone, 0.5 and 19.5ml, respectively. The aqueous phase was prepared by dissolving 50mg of the surfactant, pluronic F-68 (Sigma, St. Louis, MO, USA), in 40ml of distilled water. The organic phase was rapidly poured into the aqueous solution under stirring on a magnetic plate leading to spontaneous formation of NP. The organic solvents were evaporated, and formulation volume was adjusted to 10ml under vacuum that was gradually reduced from 180 to 12mmHg using Rotavapor (B. uchi, Switzerland) at 35ºC. Finally, the formulation was filtered through 0.45mm hydrophilic syringe filter (Sartorius, Germany).

#### NP Characterization

NP size, distribution and morphology were determined using photon correlation spectroscopy (ALV-GmBH, Langen Germany) and transmission electron microscope (TEM CM 12 Philips, Eindhoven, The Netherlands). The mean diameters obtained 223±64nm and 125±15nm, ALN and AG-1295 NP, respectively. The zeta potential was measured by means of a Zetamaster (ZEM, Malvern Instruments, Orsay, France). The zeta potential obtained was, -4.4±0.9, and –0.3±0.1mV, ALN and AG-1295 NP, respectively. The features of alendronate and AG-1295 loaded NP as well as blank NP are summarized in Table **1**.

#### Cell Internalization and Uptake of NP

ALN-NP and AG-1295 NP cellular uptake was assessed by confocal microscopy (Zeiss LSM 410, Germany). Quantitative uptake was determined by means of fluorescence activated cell sorting (FACS, Becton-Dickinson Immunocytometry Systems, USA). Fluorescent NP were prepared using BODIPY (493/503) [12] or rhodamine (530/585). A PLA-BODIPY conjugate was synthesized in our lab from 8-bromomethyl-4,4-difluoro-1,3,5,7-tetramethyl-4-bora-3a,4a-diaza-s-indacene (Molecular Probes, Inc), and PLGA-NP labeled with rhodamine was synthesized in our lab PLGA rhodamin (1:10 ratio PLGA: PLGA Rhodamine, Molecular Probes, Inc). Fifty ml of the fluorescent NP suspension was mixed with the growth medium (0.5x10^6^ cells/well), in a total concentration of 1mg/ml. Following 24hr of incubation, the cells were washed 3 times with PBS, fixed with formaldehyde (4%, 4min), and were washed another 3 times with PBS. The cells were observed using 543/570nm or 493/503nm filters. For FACS analysis, following incubation the cells were washed 3 times with PBS containing 1% FCS and were analyzed according to their relative size, side scattering and fluorescence intensity. NP with no fluorescent probe served as background for auto-fluorescence signals. Mean fluorescence intensity values were determined as linear values in the CellQuest software.

#### Statistics

All proliferation and inhibition experiments were performed in triplicates, and the data is expressed as mean±SD. Results were analyzed by Kruskal-Wallis nonparametric ANOVA test, and Dunn’s multiple comparisons post-test was calculated when the p value was less than 0.05. p<0.05 was considered significant, and p < 0.005 was considered very significant.

## RESULTS

### Cell and Tissue Culture Media

Initially we examined the influence of serum type and concentration on the optimal conditions of SMC's outgrowth from arterial explants, and their growth in cell culture. Optimal growth conditions of SMC were obtained only when serum was added (15% or 10% of medium), and when the serum used in the culture media was of the specific species. SMCs cultures of rat, rabbit and porcine species failed to replicate and to achieve a normal cell cycle in serum free medium (SFM) at all seeded densities (10,000, 30,000 and 50,000 cells/well). The addition of 5% FCS to the medium improved the proliferation rate, from no replication to a low replication rate (the cells duplicated their number on day 5, but cell number was decreased afterwards; on day 12, cell number was as the initial seeding number). The addition of 15% serum of the specific species in the rabbit, porcine, and human cultures yielded a linear growth curve, duplicating their number every 6 days. Similar growth was obtained for human cultures when the medium was supplemented with a mixture of human and FCS (7.5% each). Rat SMC were found to be the least sensitive and grew optimally in 10% FCS with no need of rat serum. All experiments were carried out afterwards with culture media supplemented with 15% serum of the specific species examined (rabbit and porcine), 10% FCS in rat's cultures, and 15% serum (7.5% human serum and 7.5% FCS) in human's cultures.

### Tissue Culture: SMC Outgrowth Rate from Arterial Explants

**Fig. (1):**
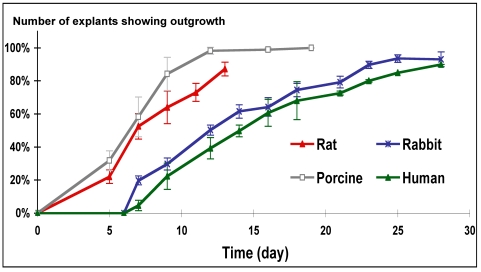
Explants outgrowth curve from various species incubated in cell culture dishes. Medium was changed every 2 days after the first week. Note that rat and porcine explants outgrowth began almost immediately after plating, whereas human and rabbit explants displayed outgrowth only by day 5 to 7.

The arterial explant model was developed in order to mimic SMC proliferation and migration from injured arteries in restenosis models [39, 40]. The cells migrating out from the explants of all species assumed the well-known hill-and-valley shape [41, 42]. In initial experiments we wished to elucidate whether outgrowth rate is species-dependent. The rat and porcine explants exhibited outgrowth almost immediately after plating, whereas human and rabbit explants exhibited a delayed outgrowth (days 5 to 7; Fig. **1**). Porcine cells were found to be the most proliferative among the species examined ranking, porcine≥rat>>rabbit>human (Fig. **1**).

### Inhibition of SMC Outgrowth

**Fig. (2):**
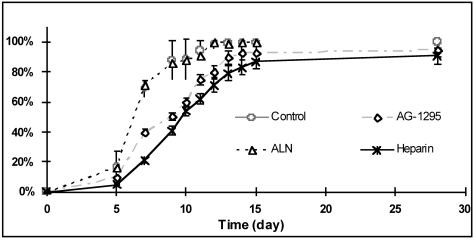
Inhibition effect of AG-1295, alendronate and heparin on SMC outgrowing from arterial porcine explants. Medium (with or without drug) was changed every 2 days after the first week.

The effect of various drugs on SMC outgrowth rate was examined on porcine arterial-explants that exhibited the fastest outgrowth rate (Fig. **2**). A marked prolongation of the time, from plating of the arterial tissue to the appearance of cells around the explants, was observed following treatment with heparin or AG-1295, but not after ALN or control treatment (Fig. **2**). Heparin, and to some lower extent, AG-1295 treatments resulted in ~50% inhibition of replication rate after 10 days. among the species examined. p<0.001 and p<0.05, porcine and human SMC vs. other species, respectively.

### Cell Culture: SMC Proliferation and Viability

**Fig. (3):**
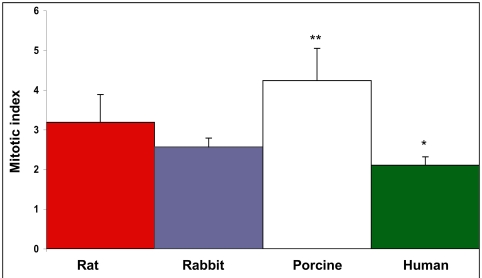
Mitotic index of SMC proliferation of various species. Cells were plated at 1.5^*^10^4^ cells per well in 24-well plates, and the rate of replication was compared 6 days following seeding. The cell replication rate was calculated as log_2_ (number of cells 6 days after plating / number of cells 24hr after plating). Porcine derived SMC were found as the most rapidly proliferating cells among the species examined. p<0.001 and p<0.05, porcine and human SMC vs. other species, respectively.

The mitotic index of SMC proliferation was determined for all species, porcine derived cells exhibited the highest mitotic rate among the species examined ranking, porcine≥rat>>rabbit>human (Fig. **3**).

**Fig. (4):**
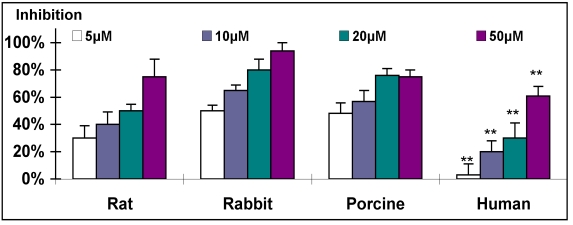
Dose response of inhibitory effect of AG-1295 on SMC from various species. For this experiment cells were plated at 2*10^4^ cells per well in 24-well plates and allowed to grow overnight. Triplicate wells were then treated with rising concentrations of AG-1295 (5- 50µM). The cells were then incubated at 37^°^C for 48hrs and counted using a Coulter counter. AG-1295 caused a marked reduction of SMC proliferation in all species. Note that rabbit and human SMC exhibited the highest and lowest sensitivity. ^**^P<0.001 human vs. other species.

**Fig. (5):**
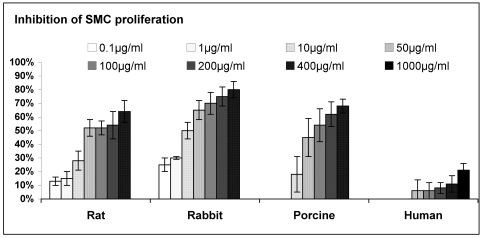
Dose response of heparin’s inhibitory effect on SMC proliferation of various species. For this experiment cells were plated at 2^*^10^4^ cells per well in 24-well plates and allowed to grow overnight. Triplicate wells were treated with rising concentrations of heparin (0.1- 1mg/ml), the cells were incubated at 37°C for 48hrs and counted using a Coulter counter. Heparin caused a marked reduction of SMC proliferation of rat, porcine and rabbit (>60%, 0.4mg/ml). Note that human SMC were not affected at those concentrations. ^**^p<0.001 human vs. other species.

Treatment of SMC of various species in cell culture with tyrphostins resulted in a marked reduction of SMC proliferation. The response to the application of a representative tyrphostin, AG-1295, is depicted in Fig. (**4**) (similar results were obtained for the other tyrphostins, AG-1296, AG-1851 and AG-2033; data not presented). A similar dose-response relationship was found for all tested species, but with different grades of sensitivity. The ranking of sensitivity was found to be: rabbit>porcine>rat>>human (Fig. **4**). As expected, heparin was found to be a potent antiproliferative drug of animal-derived SMC, with sensitivity similar to that obtained with AG-1295 treatment (Fig. **5**). No activity of heparin on human-derived SMC was observed, even at the highest concentration of 1000µg/ml (n=4); only 21% inhibition was achiev-ed in comparison to more than 50% inhibition obtained in the rat, rabbit and porcine cultures at <400µg/ml (Fig. **5**).

### Drug Delivery Systems of NP and SMC Susceptibility

**Fig. (6):**
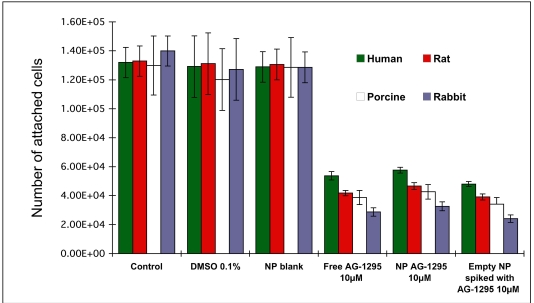
Dose response and inhibitory effect on the proliferation of SMC from various species following treatment with AG-1295 in free form, encapsulated in PLA-NP or spiked formulation (blank NP with free drug). For this experiment cells were plated at 2^*^10^4^ cells per well in 24-well plates and allowed to grow overnight. Triplicate wells were treated with rising concentrations of AG-1295 (5-50µM). The cells were incubated at 37°C for 48hrs and counted using a Coulter counter. AG-1295 caused a marked reduction of SMC proliferation in all species. Note that no significant differences were observed between the various treatments (free drug, NP, or a spiked formulation), but species sensitivity was different. DMSO in the media served as control in AG-1295 experiments.

Treatment of SMC by the tyrphostin, AG-1295, encapsulated in NP resulted in a similar degree of inhibition as the free drug in solution (68%, Fig. **6**). Empty NP spiked with the drug exhibited a similar inhibitory effect. Different sensitivity was observed among the various species, rabbit>porcine>rat>human (Fig. **6**).

**Fig. (7):**
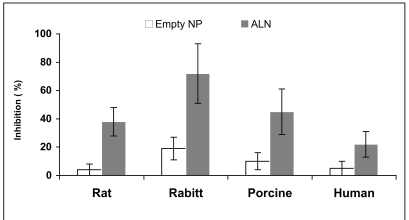
*In vitro* inhibition of SMC proliferation of various species; rat, rabbit, porcine and human, by alendronate encapsulated in NP (50µM) in comparison to blank NP. Cells were plated, allowed to grow overnight, treated with 50µM alendronate or equal amount of blank NP, incubated for 48hr, and counted using a Coulter counter and MTT assay. The grade of sensitivity obtained was identical for both treatments, rabbit>porcine>rat>>human.

Alendronate loaded NP at 50µM inhibited rat SMC proliferation by 38±10%, and porcine, human, and rabbit SMC were inhibited by 45±16, 22±9 and 72%±21, respectively (Fig. **7**). Species sensitivity was ranked, rabbit>porcine>

rat>>human. Similar results were obtained with other BP; clodronate, risedronate and ISA encapsulated in NP (data not presented). As expected, treatment of SMC with free BP had no significant inhibitory effect on SMC proliferation and viability up to 100µM regardless of the species utilized.

**Fig. (8):**
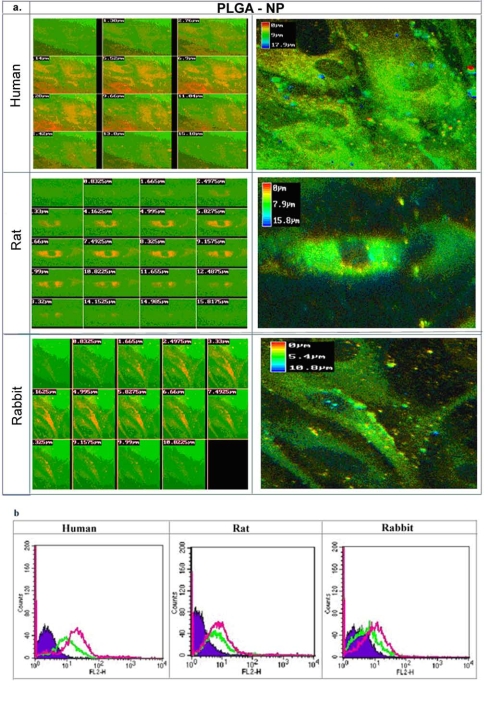
Uptake of SMC from various species (rat, rabbit, and human) incubated with fluorescently labeled PLGA-NP (Rhodamine) 24hr following treatment. a) Confocal cross-section images of SMC incubated with fluorescent NP. Note the uptake of NP, and the extensive internalization by human SMC. The NP were localized in the cytoplasm. b) Fluorescence intensity histogram of SMC detemined by FACS following application of fluorescent-labeled PLGA-NP with Rhodamin, and PLA-NP with BODIPY, 24hr after incubation. Note that intensity was higher in all species for PLGA-NP (pink) than PLA-NP (green), with extensive uptake of PLGA-NP in human SMC (60%) in comparison to 20% and 10%, rat and rabbit SMC, respectively.

In order to elucidate whether cell sensitivity is related to the different capability of the various species' SMC to internalize NP, fluorescent NP were utilized for assessing internalization. Non-labeled particles exhibited a very low fluorescent signal and were determined as background for further analyses. Confocal microscopy cross-sections verified cell internalization and the NP were localized in the cytoplasm 24hr following incubation of fluorescent-labeled PLGA-NP (Fig. **8a**). Similar experiments were carried out with PLA-NP (encapsulating tyrphostins), and a lower extent of internalization was observed in comparison to PLGA-NP (encapsulating bisphosphonates). Quantification of the uptake was determined by FACS analysis. The highest uptake of particles was observed in human SMC up to a total of 60% of the cells, and to a lower extent in SMC of rat and rabbit, 30% and 10%, respectively (Fig. **8b**). The higher internalization capacity of PLGA-NP in comparison to PLA-NP was observed in all species. The ranking of uptake between the various cell species was determined as, human>rat>rabbit.

## DISCUSSION

Typical development of new treatment modalities is cell culture studies of the chosen drug(s), animal studies in various models of restenosis, and clinical studies. Apart from pharmacokinetics and pharmacodynamic considerations [34, 43], it is expected that cell culture studies of potential antirestenotic drugs would confer the researcher with the drug of choice to be pursued in animal studies.

Initially, we established that serum of the specific species should be utilized in the culture media. Although rat SMC were found insensitive to the serum origin and could be successfully cultured with FCS, all other species SMC were optimally grown only when the same species serum was used, partially (as in human-derived tissue and cell cultures, 50% human serum with 50% FCS) or completely (as in rabbit and porcine-derived tissue and cell cultures). Moreover, when serum from different species is used misleading results could be obtained. Heparin treatment was apparently found as an effective inhibitor of SMC proliferation in human-derived SMC culture containing only FCS [20, 44], but ineffective when human serum was included in the cell culture media (Fig. **5**). This is probably because growth factors and cytokines of each species have a limited cross-reactivity.

The arterial-explant culture model is an attractive model for screening of potential antirestenotic drugs since it is apparently mimicking the tissue response to injury. Outgrowth initiation was considered as the immediate response to injury manifested by a transformed population of cells able to migrate out of the tissue and proliferate. In this model, human explants exhibited a longer time of outgrowth initiation than animal-derived explants (Fig. **1**), and the outgrowth rate was ranked as: porcine>rat>>rabbit>human. Similarly, the mitotic index obtained in SMC cultures followed the same ranking (Fig. **3**). This ranking indicates that animals' sensitivity to an antiproliferative treatment would be expected to be: porcine>rat>rabbit. This prospect is not in complete accord with the results obtained in animal studies of restenosis. The sensitivity of various animals to heparin treatment is, rat>rabbit>>porcine [15, 23, 24, 29, 45, 46]. In addition, the same ranking of animals’ sensitivity for tyrphostins treatments in restenosis models is observed [12, 33, 34, 39, 47]. Moreover, heparin was found somewhat more potent than AG-1295 in the porcine arterial explant model (Fig. **3**). Nevertheless, when those antiproliferative drugs were tested in the porcine model of restenosis, AG-1295 [ 39] and other tyrphostins [ 12,  33] exhibited significant efficacy, whereas mixed results were reported for heparin's efficacy [ 23,  24,  29].

The anticipated sensitivity ranking from the explant outgrowth model and SMC mitotic rates are not in accordance with the observed relative sensitivity of various animal species to antiproliferative therapy in restenosis models. On the other hand, it is expected from the arterial explant outgrowth model that the human (Fig. **1**) would be the least sensitive to an antiproliferative treatment. Indeed, heparin was found ineffective in clinical trials [ 16,  18,  19].

As can be evidenced, heparin, even at a high concentration of 1mg/ml, exhibited no antiproliferation effect on human SMC (Fig. **5**), in accord with heparin's inactivity in human trials [ 16, 18,  19]. In addition, the profound antiproliferative activity exhibited by AG-1295 on SMC of various animal species has been reported to be successfully reproduced *in vivo* [ 34,  39]. The ranking of species sensitivity in SMC culture to AG-1295 and heparin was rabbit>porcine>rat>>human (Figs. **4**-**5**). The rabbit and porcine SMC were the most sensitive. The reason(s) underlying this ranking of activity is unknown and beyond the scope of this work. Genetic variability could explain the variability in response to different drugs among species as was suggested for SMC cell types of different responsiveness [48]. It should be noted that the above ranking of sensitivity is not inversely correlated to the mitotic index (porcine>rat>rabbit>human, Fig. **3**). The mitotic index could represent the relative age of the species (juvenile pigs on one hand and old humans on the other, rats and rabbits in-between). It is suggested that prescreening studies of possible drug candidates for restenosis therapy should include both SMC cell cultures of rat and human, appropriately designed with a suitable serum. Moreover, examination of possible candidates for restenosis inhibition in cell culture models could be performed in rat and human SMC cultures, and not necessarily in the same species intended to be examined *in vivo*. It should be recalled that both taxol and sirolimus, utilized clinically in DES, exhibited profound inhibition of porcine as well as human-derived SMC in culture [ 49].

It remains unclear whether any single restenotic animal model is of a more predictive value than others to the human response, and what species is a better predictor for a specific antirestenotic treatment [1, 46,  50]. Stenting of porcine coronary artery or the rabbit iliac artery are the most frequently used animal models for in-stent restenosis [45, 51].

*In vivo* studies in the rat and porcine models revealed a rapid biodegradation of AG-1295 when administered in solution [ 30,  39]. Encapsulation of tyrphostins in NP and polymeric matrices was shown to maintain drug stability, with a sustained release profile that matches the time course of the pathologic processes [ 31]. However, the significant advantage of NP over free drug could not be detected *in vitro* (Fig. **6**). This is probably because there is neither drug wash out nor biodegradation as occurs *in vivo*, and internalization of the NP parallels the efficient transport of the hydrophobic tyrphostin into the cells.

Although SMCs aren’t phagocytic, they posses the capability to uptake and internalize polymeric nanoparticles [12]. The preferential uptake of PLGA-NP in comparison to PLA-NP by SMC by all species (rat, rabbit and human, Fig. **8b**) could be explained by the negatively charged surface of PLGA vs. the neutral charge of PLA NP [ 52,  53]. It is well known that charged particles are avidly internalized by phagocytic cells [ 54,  55]. It is plausible to assume that the adsorbed proteins on the negatively charged NP contributed to internalization.

*In vivo* results obtained in our group have shown that treatment with BP in free form or encapsulated in NP, in the rat and rabbit models of restenosis, do not influence SMC directly [ 35- 37,  56]. The reduction of cell proliferation is attributed to decreased inflammation response in the artery, following inhibition of circulating monocytes and depletion of arterial macrophages [ 35- 37,  56]. It is interesting to note that NP internalization followed the ranking of: human>rat>rabbit (Fig. **8**), but rabbit-derived SMC were found as the most sensitive to ALN-NP treatment (Fig. **7**). It is suggested that the unique sensitivity of rabbit SMC detected in the SMC culture studies (Fig. **7**) contributed to the profound effect of ALN-NP rather than their internalization capability. Taken together, the similar effects exerted by AG-1295 NP, free drug and spiked NP (Fig. **6**) and the effects of ALN NP (Fig. **7**), it can be concluded that the validity of *in vitro* culture studies for screening controlled release delivery systems such as NP is limited.

## CONCLUSIONS

Reliable SMC culture studies can be obtained if the serum of the specific species is utilized in the culture media. It is advisable to examine a new drug candidate in the rat and human SMC cultures. Prescreening of possible drug candidates for restenosis therapy should always include studies in human-derived SMC culture, which included human serum.

## Figures and Tables

**Table 1.** The Physicochemical Properties of Nanoparticles Formulations Utilized in the Efficacy Studies

** Formulation**	**Blank NP**		**ALN-NP**	** Tyrphostin NP**
	**PLGA**	**PLA**		
Entrapment (%) (% of initial)			55.1±7.4	70.8±4.1
Particle size (nm)	215±53	121±13	223±64	125±15
Zeta potential (mV)	-2.5±0.7	-3.5±0.5	-4.4±0.9	-0.3±0.1
